# Molecular sex identification of Malaysian White‐Nest Swiftlet (*Aerodramus fuciphagus* Thunberg, 1812)

**DOI:** 10.1002/ece3.6699

**Published:** 2020-08-25

**Authors:** Muhammad Amin Osman, Sumita Sugnaseelan, Jothi Malar Panandam, Nurul Izza Ab Ghani

**Affiliations:** ^1^ Department of Animal Science Faculty of Agriculture Universiti Putra Malaysia Serdang Selangor Malaysia; ^2^ Ecotone Worldwide Sdn. Bhd Kelana Centre Point Petaling Jaya Selangor Malaysia; ^3^ Department of Biology Faculty of Science Universiti Putra Malaysia Serdang Selangor Malaysia

**Keywords:** *Aerodramus fuciphagus*, CHD gene, molecular sexing

## Abstract

The difficulty in differentiating the sex of monomorphic bird species has made molecular sexing an important tool in addressing this problem. This method uses noninvasively collected materials such as feathers and may be advantageous for sexing endangered as well as commercialized bird species. In this study, seven primer sets for sexing birds were screened in *Aerodramus fuciphagus* using a total of 13 feather samples that were randomly selected from the state of Perak, Malaysia. From the screening analysis, only one primer set (P8/WZ/W) successfully differentiated the sex of *A. fuciphagus*. PCR amplification produced a single 255‐bp DNA fragment for males which was derived from *CHD‐Z* (CHD gene region in the sex chromosome Z), while for the females it produced two fragments (144 and 255 bp). The 144‐bp fragment was from *CHD‐W* (CHD gene region in the sex chromosome W). Results from sequencing showed no variations in the base sequences of the *CHD‐W* and *CHD‐Z* amplified fragments within the same sexes, except for one male sample (A23) where at position 166, a base substitution occurred (G → A). Phylogenetic analysis of *CHD‐W* showed that four (Apodiformes; Gruiformes; Passeriformes; and Pelecaniformes) out of the five orders investigated had formed four clear clusters within their orders, including the studied order: Apodiformes. Whereas in *CHD‐Z*, four (Accipitriformes; Columbiformes; Galliformes; and Passeriformes) out of five orders investigated formed four clear clusters within their orders, excluding the studied order. In addition, *A. fuciphagus* and *Apus apus* (both Apodiformes) showed less divergence in *CHD‐W* than *CHD‐Z* (0% *c.f*. 9%). The result suggests that in *A. fuciphagus*, CHD gene evolution occurred at a higher rate in males (*CHD‐Z*) compared to females (*CHD‐W*). This finding may be useful for further studies on sex ratio and breeding management of *A. fuciphagus*.

## Graphical abstract

In this study, a molecular sexing method was used to identify the sex of monomorphic and economically important bird species; *Aerodramus fuciphagus* (Thunberg, 1812). Interestingly, we found one male sample (A23) where at position 166, had a base substitution (G → A), hence, an evolution study in CHD‐W and CHD‐Z genes by comparing *A. fuciphagus* with 14 other bird species from the same Apodiformes order (*Apus apus* and *Calypte costae*) and Aves class was performed. This study is important for further studies on sex ratio and breeding management of *A. fuciphagus* as well as CHD gene evolution.

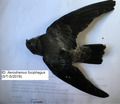

## 1 INTRODUCTION

Individuals’ sex information is important in the field of evolutionary biology, breeding, and conservation (Fridolfsson & Ellegren, 1999). Animals may be sexed using morphological, surgical, cytological, and molecular methods. Among these methods, molecular sexing is considered as the most accurate method (Dubiec & Zalagska‐Neubauer, 2006), especially for sexing animals that lack sexual dimorphism, such as most birds. Molecular sexing in birds is based on PCR amplification of alleles on Z and W chromosomes using specific primers designed to screen variations in length and nucleotide sequence (Morinha, Cabral, & Bastos, 2012). Female birds are heterogametic (ZW) while males are homogametic (ZZ). Over the years, numerous molecular sexing markers have been designed to accurately sex birds. However, due to the diverse species of birds investigated, only a few molecular sexing markers were able to successfully differentiate the sex of these birds.

Molecular sexing in birds was initiated by Griffiths and Tiwari (1995). They discovered the W‐linked gene that was later identified for encoding a chromo‐helicase‐DNA‐binding protein (*CHD*) which found to present in most birds (Ellegren, 1996; Griffiths, Daan, & Dijkstra, 1996), and related to human *CHD1* (Woodage, Basrai, Baxevanis, Hieter, & Collins, 1997). A related Z‐linked gene, *CHD‐Z,* had been reported by Griffiths and Korn (1997). Griffiths et al. (1996) proved that this gene is remarkably conserved, and a single set of PCR primer may be used for molecular sexing in birds throughout the class Aves, with the exception of ratites. This primer simultaneously amplifies the homologous parts of *CHD‐W* and *CHD‐Z*. The two amplified *CHD* products were of the same size but may be differentiated using restriction enzyme which selectively cuts the *CHD‐Z* DNA fragment, generating two electrophoretic bands for the females and only one for the males. To overcome the problem of the same PCR product sizes for *CHD‐W* and *CHD‐Z*, Griffiths, Double, Orr, and Dawson (1998) proposed a protocol for sexing of birds using two PCR primers which anneal at the conserved exonic region and amplified across an intron, which being noncoding and less conserved, varying in length between *CHD‐W* and *CHD‐Z*.

Fridolfsson and Ellegren (1999), however, claimed that the primers suggested by Griffiths et al. (1998) were not always successful in differentiating *CHD‐W* and *CHD‐Z* fragments by using standard agarose electrophoresis, and therefore, proposed another set of primer which consistently amplified different sizes of DNA fragments for *CHD‐W* and *CHD‐Z* in the majority of bird species. This particular pair of primer (2550F and 2718R) was designed based on the constant size differences between *CHD1W* and *CHD1Z* introns. Using highly conserved pair of primer flanking the intron, PCR amplification, and agarose gel electrophoresis, female birds displayed either one (*CHD1W*) or two fragments (*CHD1W* and *CHD1Z*), while males showed a single fragment (*CHD1Z*) with a clear difference in size from the female‐specific *CHD1W* fragment. From the 50 bird species studied, 47 species successfully amplified the *CHD1W* fragment between 400 and 450 bp in size and *CHD1Z* fragment between 600 and 650 bp. Later, an almost similar method was designed by Kahn, St. John, and Quinn (1998) which based on two highly conserved regions that flanked the intervening introns, common to both Z‐linked and W‐linked CHD genes. From it, a pair of primer was synthesized (1237L and 1272H). The sizes of amplification products for this pair of primer differ over a range of 210–285 bp for each bird species (total of 17 bird species investigated), and the size differences between the W‐ and Z‐specific copies of this amplification also vary among those bird species. Thus, some of the bird species showed a very small size difference such as found in Red‐tailed Hawk *(Buteo jamaicensis*) and Great Horned Owl (*Bubo virginiasus*). Furthermore, in some other bird species, the size differences cannot be resolved on standard agarose gel electrophoresis and needed the use of non‐denaturing polyacrylamide gel electrophoresis method.

The most widely used molecular sexing method for birds usually involves the exploitation on size differences between introns of the CHD gene on the Z and W sex chromosomes (Fridolfsson & Ellegren, 1999; Kahn et al., 1998). Heterogametic (ZW) females are expected to have two different sized introns while homogametic (ZZ) males should only have one intron size. However, the existence of polymorphism in the introns of *CHD‐Z* in several species has complicated the identification of sex because heterozygote (e.g., ZZ’) males will also have two different sized introns (Dawson et al., 2001; Lee, Brain, Forman, Bradbury, & Griffiths, 2002; Robertson & Gemmell, 2006). Due to this concern, another molecular sexing primer set to improve the reliability in birds sexing had been introduced by amplifying an additional W chromosome‐specific DNA fragment of a different size than that produced by the pre‐existing sexing primer pairs to independently confirm the existence of the W chromosome. Therefore, Shizuka and Lyon (2008) developed a reverse primer (GWR2) designed to sit within the intron of the W chromosome and amplify a small DNA fragment that served as a W‐specific marker. This primer combines with the primer pair (1237L and 1272H) developed by Kahn et al. (1998) and subsequently used to amplify the genes of the individual birds from American coots’ species (*Fulica americana*). It was essentially a multiplex reaction of two primer pairs, the 1237L/1272H that amplified the entire *CHD1* intron of both Z and W chromosomes, and 1237L/GWR2 pair that amplified a short fragment when the W chromosome was present. The other recent molecular sexing in bird species was conducted by Hagadorn, Tell, Drazenovich, and Ernest (2016)which had evaluated 11 primer pairs from CHD region on sex chromosomes in hummingbird and obtained only two primer sets (P8/P2 and 1237L/1272H) resulted in reliable DNA amplification to determine sex in this species of bird. Therefore, molecular sexing for determining sex in avian species is important to accurately identify sex for sustainable species management (e.g., breeding) and provides vital data for comparative genomic studies such as sex evolutionary.

**Figure 1:**
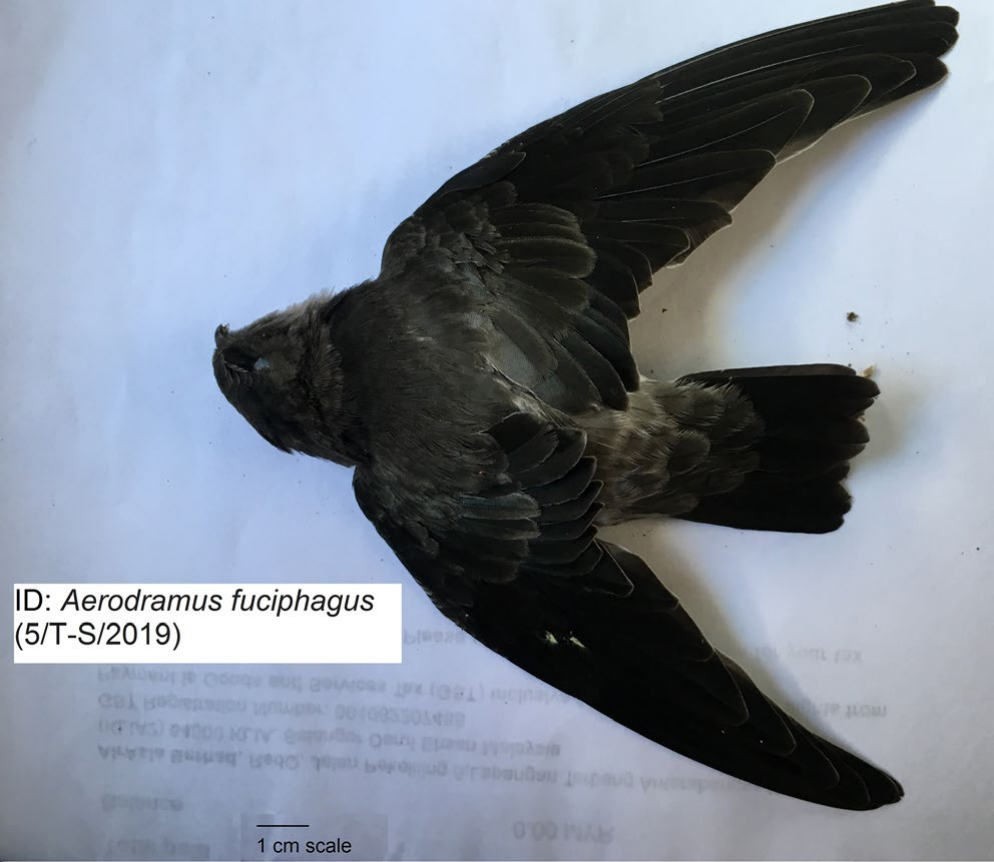
Malaysian White‐Nest Swiftlet (Aerodramus fuciphagus Thunberg, 1812)

Though many studies on molecular sexing in birds had been conducted, but no study for *Aerodramus fuciphagus* (White‐nest swiftlet; Figure 1) which is a bird with lack of sexual dimorphism has been done. This bird species showed no distinguishable external feature or behavior between male and female which make it hard to differentiate their sex using morphological method. It is important to be able to differentiate males from females of *A. fuciphagus* for sustainable breeding, to ensure population viability and growth of this economically important swiftlet species which produces nests that are edible (Lim, 2011). The nests have high commercial values because they are made from pure saliva of the birds (Kang & Lee, 1991) and contain ~8.6% sialic acid (a major component of glycoprotein) which is considered of high medicinal and nutrient benefits (Chau et al., 2003; Kathan & Weeks, 1969; Vimala, Hussain, & Nazaimoon, 2012). Nutrients in the nests have been claimed to be beneficial for reducing the risk of cardiometabolic conditions associated with estrogen deficiency such as diabetes and cardiovascular disease (Hou, Imam, Ismail, Ooi, et al., 2015). EBN may also possess anti‐inflammatory (Vimala et al., 2012) and antioxidant properties (Hou, Imam, Ismail, Azmi, et al., 2015). Furthermore, EBN has been suggested to be effective for the improvement of bone loss (Matsukawa et al., 2011) and it may be a potential antidegenerative agent for treating osteoarthritis (Chua et al., 2013). Therefore, this study aims to identify molecular sexing markers for *A. fuciphagus* and compare the amplified *CHD‐W* and *CHD‐Z* sequences with other bird species.

## 2 MATERIALS AND METHODS

### 2.1 Sample collection and PCR protocol

**Figure 2:**
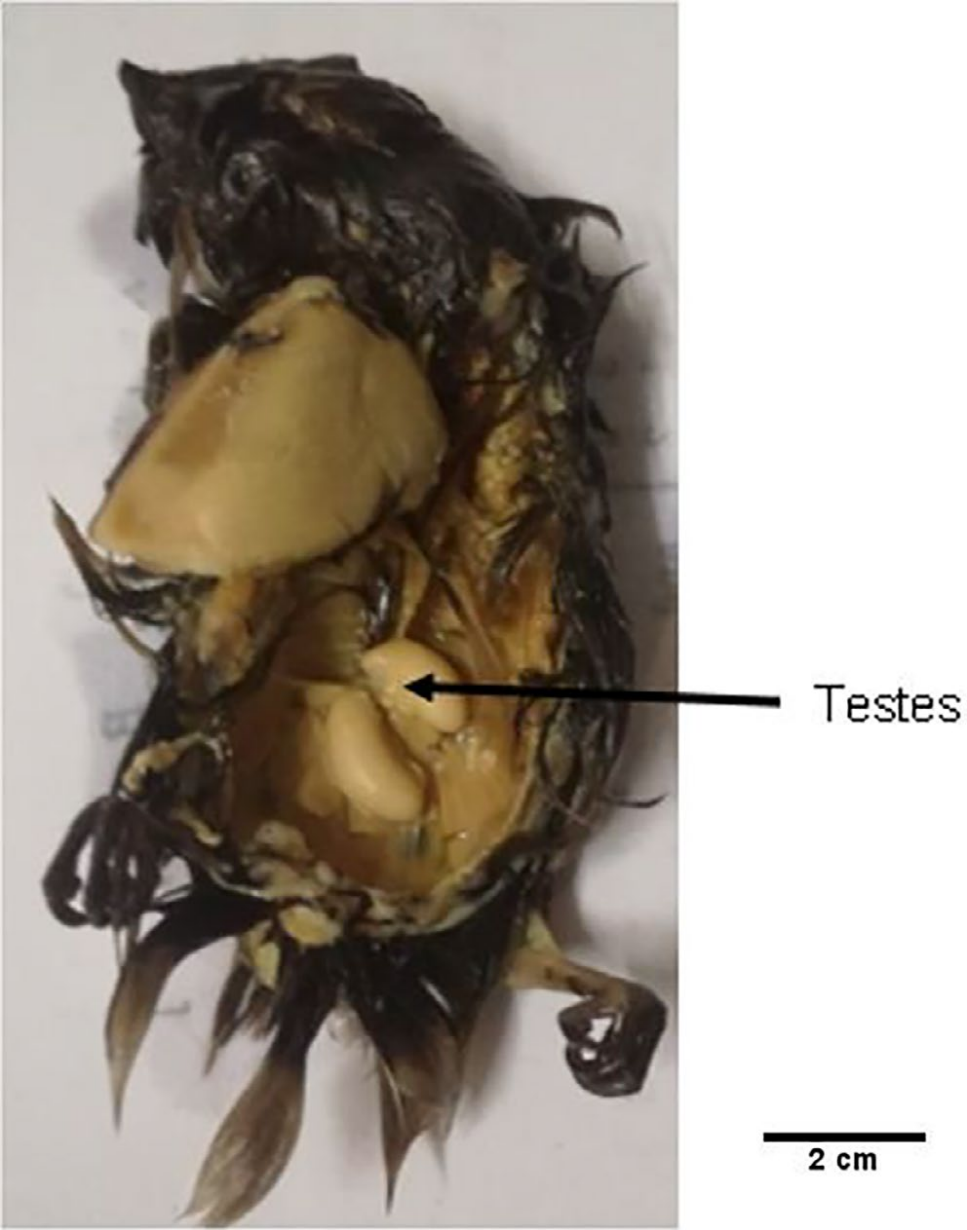
Male *A. fuciphagus* (M1, M2, and M3) determined by postmortem

In an attempt to obtain samples of male and female swiftlets, a postmortem was conducted on 13 randomly selected *A. fuciphagus* carcasses from Northern Region (Perak) swiftlet houses to positively confirm the sex of each individual *via* gross visual identification of the sex gonads. From the 13 individual carcasses dissected, the sex gonads could be identified in only three of the carcasses. Based on the distinct testes identified, these three carcasses were confirmed as male *A. fuciphagus* (Figure 2) and were used as control (M1, M2, and M3) in the subsequent investigation for molecular sexing. On the other hand, feather samples collected from 10 unknown sex of dead chicks or chicks that had dropped from their nests at the breeding colonies were used as candidate samples to screen for the sexing markers. Hence, the electrophoresis banding patterns for the 10 candidate samples (unknown sex) were compared to those of the controls. Samples showed similar patterns as the confirmed males were identified as male individuals, and samples showed banding patterns that differed from the controls were assumed to be female individuals.

All thirteen *A. fuciphagus* individuals including three males (determine by postmortem) and 10 unknown sex were used as samples in this molecular sex identification study. All samples were taken from the calamus tip of a primary feather and extracted for DNA using DNeasy^®^ Blood & Tissue Kit (Qiagen, Hilden, Germany) by following the manufacturer's protocol. A total of seven different molecular sexing primers were used in this study (Table 1). Primers P8/P2/P0, P8/WZ/W, and 1237L/1272H/GW2 used the multiplex PCR method. PCR was carried out in a total volume of 25 μl containing 5 µl of 5 × PCR buffer, 50 ng/µl of genomic DNA, 1 unit of Taq polymerase (Promega Corporation), 1.5 mM MgCl2, 1 µM of each primer, 0.2 mM of dNTPs, and distilled water. Touchdown PCR protocol for all samples using primer set of P8/WZ/W was as follows: initial denaturation at 95°C for 5 min, followed by 10 cycles of 95°C denaturation for 30 s, 60°C to 50°C (decrease temperature by 1°C in each cycle) annealing for 30 s, and 72°C extension for 45 s, the last 30 cycles maintain the annealing temperature of 50°C before proceeded with a final extension at 72°C for 5 min. PCR products were separated by electrophoresis on 3% agarose gel and visualized under ultraviolet light to observe the PCR amplicon bands.

**Table 1:** Seven sex primers tested for identifying the sex of *A. fuciphagus*

Forward Primer	F‐sequence	Reverse Primer	R‐sequence
P8^a^	5′‐CTCCCAAGGATGAGRAAYTG‐3′	P2^a^	5′‐TCTGCATCGCTAAATCCTTT‐3′
P0^a^	5′‐ATTGAGTTGGAACCAGAICA‐3′		
1237L^b^	5′‐GAGAAACTGTGCAAAACAG‐3′	1272H^b^	5′‐TCCAGAATATCTTCTGCTCC‐3′
P8^c^	5′‐CTCCCAAGGATGAGRAAYTG‐3′	WZ^c^	5′‐CCCTTCACTTC CATTAAAGC‐3′
		W^c^	5′‐ACCCAACCCAAAAGTACAAG‐3′
1237L^d^	5′‐GAGAAACTGTGCAAAACAG‐3′	1272H^d^	5′‐TCCAGAATATCTTCTGCTCC‐3′
		GW2^d^	5′‐CCTGTAAAAACCACCCAACC‐3′
2550F^e^	5′‐GTTACTGATTCGTCTACGAGA‐3′	2718R^e^	5′‐ATTGAAATGATCCAGTGCTTG‐3′
P8^f^	5′‐CTCCCAAGGATGAGRAAYTG‐3′	P2^f^	5′‐TCTGCATCGCTAAATCCTTT‐3′
P8^g^	5′‐CTCCCAAGGATGAGRAAYTG‐3′	M5^g^	5′‐YTYMCTTCAYTTCCATTAAAGC‐3′

^a^ Original primer design from Han, Kim, Kim, Park, and Na (2009).

^b^ Original primer design from Kahn et al. (1998).

^c^ Original primer design from Wang et al. (2011).

^d^ Original primer design from Shizuka and Lyon (2008).

^e^ Original primer design from Fridolfsson and Ellegren (1999).

^f^ Original primer design from Griffiths et al. (1998).

^g^ Original primer design fromBantock, Prys‐Jones, and Lee (2008)

### 2.2 Nucleotide sequence analysis

PCR products that showed variations in genotypic patterns were chosen for further investigation. The PCR products were purified and sent for sequencing to First Base Laboratories Sdn Bhd, Selangor. One male control (sex determine by post‐mortem) together with two expected male and two expected female samples (identified as such from banding patterns) were selected for sequencing (a total of five samples). The two expected male and one male control samples displayed only a single band pattern on electrophoresis, and therefore, the PCR products were purified using PCR purification method. Meanwhile, the two expected female samples showed two band patterns on electrophoresis, the PCR product bands of interest (144‐bp band) were visualized with the help of UV transilluminator and excised from the agarose gel using a sterile scalpel and transferred into a 1.5‐ml microcentrifuge tube and weighed before purified using gel purification method. All selected samples were purified using Geneall^®^ ExpinTM Combo GP (Geneall Biotechnology, Korea) by following the manufacturer's protocol.

### 2.3 Data analysis

The nucleotide sequences of representative males and females of *A. fuciphagus* were aligned and compared individually using BLASTN (National Centre of Biotechnology Institute, NCBI) with the sequence of a control individual. Similarity in the nucleotide sequences among the males, females, and control were compared and the percent identity was estimated using Clustal Omega software (http://www.ebi.ac.uk/Tools/msa/clustalo) to confirm the sex of the *A. fuciphagus* and the CHD‐W and CHD‐Z genes. The nucleotide sequences of males and females of *A. fuciphagus* were aligned and compared individually using BLASTN, NCBI, with a homologous nucleotide sequence of 14 other bird species from the same Apodiformes order (*Apus apus* and *Calypte costae*), and Aves class to understand the evolution of CHD gene. Then, phylogenetic trees for *A. fuciphagus* and 14 other bird species were constructed using MEGA 7 (Kumar, Stecher, & Tamura, 2016) using neighbor‐joining method (NJ) (Saitou & Nei, 1987) with 1,000 bootstrap.

## 3 RESULTS

**Figure 3:**
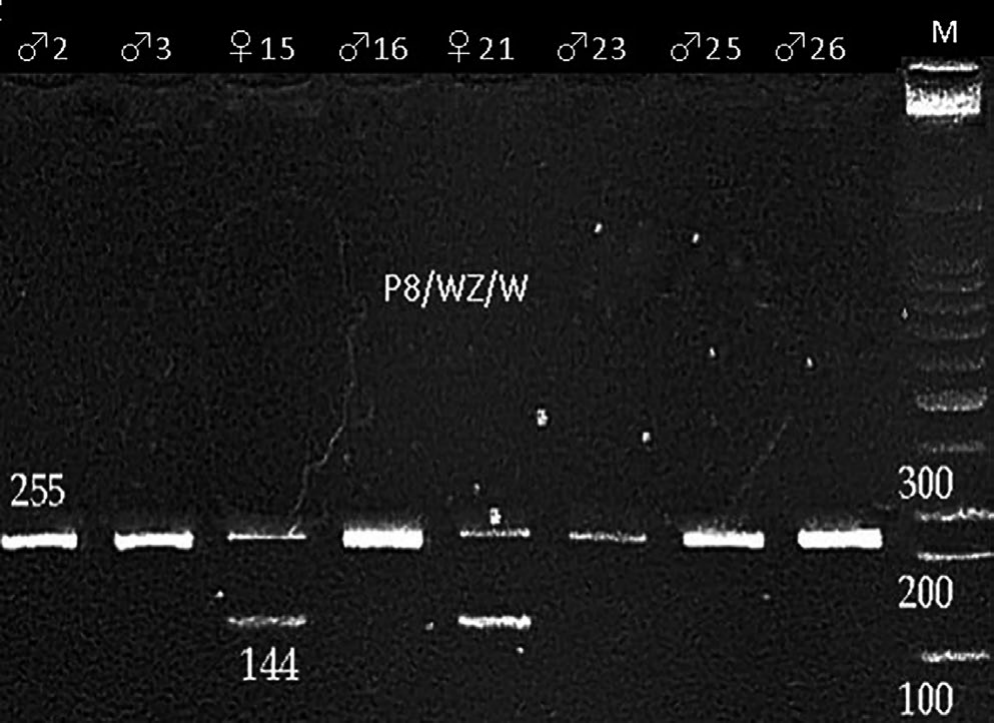
Agarose gel showing PCR amplicons from an optimized PCR composition and protocol by P8/WZ/W sexing primer set for eight swiftlet samples (♂2 and ♂3: control male 2 and 3; ♀15 and ♀21: possible female; ♂16, ♂23, ♂25, and ♂26: possible male; M: 100 base pair DNA ladder)

In this study, seven avian sexing marker sets based from *CHD* region on sex chromosome in birds had been screened and results showed that only one sexing marker set (P8/WZ/W) could successfully differentiate males from females *A. fuciphagus* (Figure 3). The results from this study showed the presence of CHD‐Z gene (255 bp; Figure 2) on the control males (M2 and M3) and possible male samples (A16, A23, A25, and A26) while possible female samples (A15 and A21) showed the presence of extra band, CHD‐W gene (144 bp; Figure 2). In order to determine the validity of the P8/WZ/W sexing primer set for identifying the sex of *A. fuciphagus*, PCR products of two candidate male samples (A16 and A23) and excision of amplified bands from an agarose gel of two candidate female samples (A15 and A21), together with PCR products of a control male sample (M3) were purified and sent for sequencing. The obtained sequences were submitted to GenBank with accession numbers MH445397 (M3), MH445398 (A15), MT591397 (A16), MT591398 (A23), and MT591399 (A21), respectively. Sequencing results showed no variation within the same sexes for both males and females except for one male sample (A23), which at base position 166 exhibited a base substitution from G (guanine) to A (arginine). This suggests that point mutation had occurred in CHD‐Z gene by the exchange of one base to another.

**Figure 4:**
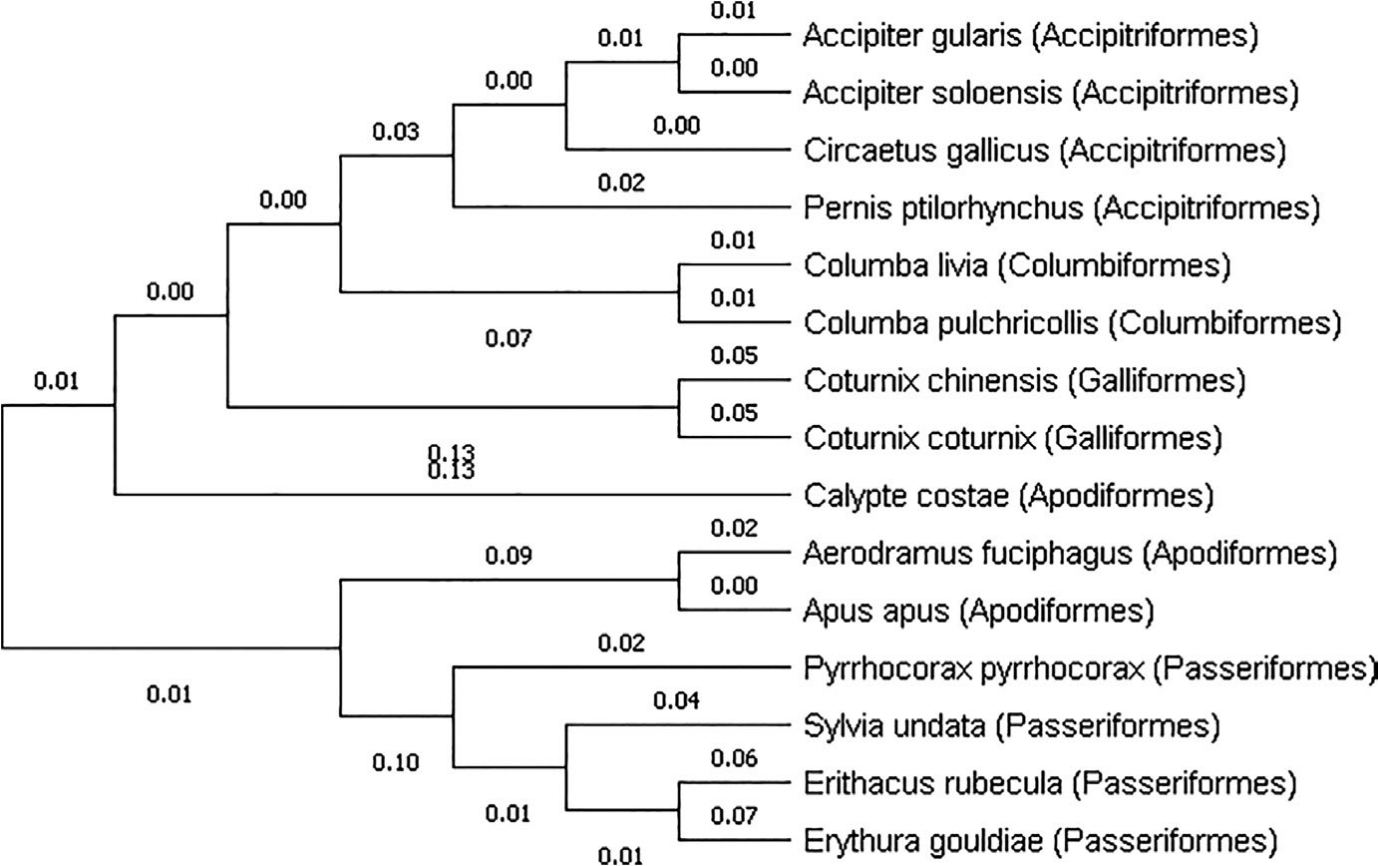
Neighbor‐joining tree based on DNA genetic distances (Nei's standard genetic distances) of CHD‐Z gametolog showing the genetic relationships between male *A. fuciphagus* and male birds of the same (Apodiformes) and different order (Accipitriformes, Columbiformes, Galliformes, and Passeriformes)

**Figure 5:**
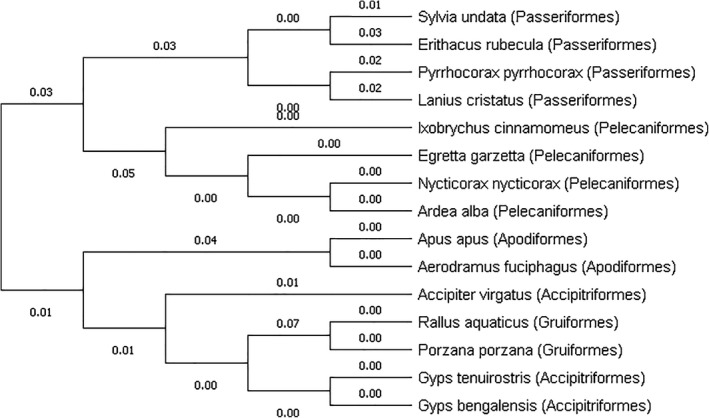
Neighbor‐joining tree based on DNA genetic distances (Nei's standard genetic distance) of CHD‐W gametolog showing the genetic relationships between female *A. fuciphagus* and female birds of the same (Apodiformes) and different order (Accipitriformes, Gruiformes, Passeriformes, and Pelecaniformes)

The sex evolution of *A. fuciphagus* in the present study with respect to other avian species was investigated based on the percentage of similarity within the CHD gene loci of *A. fuciphagus* compared to 14 other avian species. Male *A. fuciphagus* showed a percentage of similarity ranged from 74% to 98% with other male birds (*CHD‐Z*) of the Apodiformes order or Aves class (Table 2). Female (*CHD‐W*) *A. fuciphagus* on the other hand showed the percentage of similarity with other female birds of the Apodiformes order or Aves class ranging from 83% to 100% (Table 3). The results of phylogenetic analysis of *A. fuciphagus* including 14 other bird species from either the same Apodiformes order or same Aves class for both *CHD‐Z* (male) and *CHD‐W* (female) genes also showed that there were sex evolutionary patterns in *CHD* region on sex chromosomes among bird species (Figures 4 and 5).

**Table 2:** Percentage of sequence similarity between male *A. fuciphagus* with other birds of the same Apodiformes order or Aves class

Reference Male	Species and Common Name	Percentage of sequence similarity (%)	Order
*A. fuciphagus*	*Apus apus* (Common swift)	98	Apodiformes
*A. fuciphagus*	*Calypte costae* (Costa's hummingbird)	80	Apodiformes
*A. fuciphagus*	*Pernis ptilorhynchus* (Crested honey buzzard)	82	Accipitriformes
*A. fuciphagus*	*Accipiter gularis* (Japanese sparrow hawk)	81	Accipitriformes
*A. fuciphagus*	*Accipiter soloensis* (Chinese sparrow hawk)	81	Accipitriformes
*A. fuciphagus*	*Circaetus gallicus* (Short‐toed snake eagle)	81	Accipitriformes
*A. fuciphagus*	*Sylvia undata* (Dartford warbler)	76	Passeriformes
*A. fuciphagus*	*Pyrrhocorax pyrrhocorax* (Red‐billed chough)	77	Passeriformes
*A. fuciphagus*	*Erythura gouldiae* (Gouldian finch)	76	Passeriformes
*A. fuciphagus*	*Erithacus rubecula* (European robin)	75	Passeriformes
*A. fuciphagus*	*Coturnix coturnix* (Common quail)	74	Galliformes
*A. fuciphagus*	*Coturnix chinensis* (King quail)	75	Galliformes
*A. fuciphagus*	*Columba livia* (Rock dove)	81	Columbiformes
*A. fuciphagus*	*Columba pulchricollis* (Ashy wood pigeon)	80	Columbiformes

**Table 3:** Percentage of sequence similarity between female *A. fuciphagus* with other birds of the same Apodiformes order or Aves class

Reference Female	Species and Common Name	Percentage of sequence similarity (%)	Order
*A. fuciphagus*	*Apus apus* (Common swift)	100	Apodiformes
*A. fuciphagus*	*Gyps tenuirostris* (Slender‐billed vulture)	91	Accipitriformes
*A. fuciphagus*	*Gyps bengalensis* (White‐rumped vulture)	91	Accipitriformes
*A. fuciphagus*	*Accipiter virgatus* (Besra)	91	Accipitriformes
*A. fuciphagus*	*Lanius cristatus* (Brown shrike)	87	Passeriformes
*A. fuciphagus*	*Pyrrhocorax* (Red‐billed chough)	87	Passeriformes
*A. fuciphagus*	*Sylvia undata* (Dartford warbler)	86	Passeriformes
*A. fuciphagus*	*Erithacus rubecula* (European robin)	83	Passeriformes
*A. fuciphagus*	*Egretta garzetta* (Little egret)	83	Pelecaniformes
*A. fuciphagus*	*Ixobrychus cinnamomeus* (Cinnamon bittern)	83	Pelecaniformes
*A. fuciphagus*	*Nycticorax nycticorax* (Black‐crown night heron)	83	Pelecaniformes
*A. fuciphagus*	*Ardea alba* (Great egret)	83	Pelecaniformes
*A. fuciphagus*	*Rallus aquaticus* (Water rail)	87	Gruiformes
*A. fuciphagus*	*Porzana porzana* (Spotted crake)	87	Gruiformes

## 4 DISCUSSION

Through gross visual identification of the sex gonads, only three out of 13 individual carcasses were successfully identified as male *A. fuciphagus* (Figure 1). The reason for a failure to determine the sex of the other carcasses may be due to the various stages of decomposition of the collected carcasses. The carcasses were collected during the visits of the research team to the birdhouses. Meanwhile, the carcasses may be of juvenile individuals in which the sex organs may not yet be fully developed.

In this study, only one sexing marker set (P8/WZ/W) from seven avian sexing marker sets based from *CHD* region on sex chromosome in birds could successfully differentiated males from females *A. fuciphagus* (Figure 3). This may attribute to the fact that specificity in the expression of CHD gene among different species of birds. The same had been reported by Hagadorn et al. (2016) while screening eleven sexing marker sets from *CHD* region on sex chromosomes in hummingbird, and only obtained two primer sets (P8/P2 and 1237L/1272H) resulted in consistent DNA amplifications, and reliably determined the sex of the bird. Similar results had been reported on the most commonly used primers for PCR‐based amplification of CHD gene (P2/P8 primer pair) that proved to be useful for sex identification in 17 species but not for three species of Hawaiian birds (Jarvi & Farias, 2006). The sexing marker set P8/WZ/W that had successfully identified the sex of *A. fuciphagus* in this study was designed based on conserved regions inside the amplified P2/P8 primer pair sequences. This PCR primer set had been used for sex identification of Chinese egret and nine other ardeid species (Wang, Zhou, Lin, Fang, & Chen, 2011). The sizes of the amplified bands in this study did not show much differences from the results of the Chinese egret and nine other ardeid species for both CHD‐Z (250 bp) and CHD‐W (140 bp) genes (Wang et al., 2011). Wang et al. (2011) reported that there were some advantages in this method compared with the use of P2/P8 primer‐based technique. First of all is the fact that only one PCR was required, and results were readily analyzed by agarose gel electrophoresis without the need for tedious polyacrylamide gel electrophoresis method. Moreover, the lengths of PCR products with this primer sets were shorter (140/250 bp), compared with 380 bp using the P2/P8 (primer sets of ardeid species), which means that even degraded DNA samples may be used, making it possible to utilize noninvasive sample materials such as shed feathers that provide less DNA source materials (Harvey, Bonter, & Stenzler, 2006) for sex identification.

Furthermore, sequencing results showed no variation within the same sexes for both males and females except for one male sample (A23), which at base position 166 exhibited a base substitution from G (guanine) to A (arginine). This suggests that point mutation had occurred in CHD‐Z gene through the exchange of one base to another. The implications of such mutation can either be negligible, or in some cases a serious effect. Changes to the codon due to base substitution may encode different amino acids and cause a change in the protein produced. In some serious cases, the base substitution may change the codon to a “stop” codon and causes an incomplete protein. However, in the case where changes of codon encode the same amino acid, there will be no changes to the protein produced. This is known as a silent mutation. In this study, it seems that base substitution at CHD‐Z gene may be a silent mutation as the effect is negligible.

In addition, the sex evolution of *A. fuciphagus* in the present study with respect to 14 other avian species was investigated. These 14 avian species were chosen because they were either members of the same Apodiformes order or Aves class and have high compatibility and similarity with CHD gene from the genome database. The nucleotide sequences for *A. fuciphagus* (M3 for *CHD‐Z* and A15 for *CHD‐W*) were aligned and compared with both the *CHD‐Z* and *CHD‐W* regions of other birds. The results of sequence similarity analysis showed a high percentage of sequence similarity for both female (100%) and male (98%) *A. fuciphagus* with *Apus apus* (common swift). It is expected as both species are closely related because both are in the same Apodiformes order. However, interestingly the result of sequence similarity between *A. fuciphagus* and *Calypte costae* (Costa's hummingbird) showed a low sequence similarity for male (80%), and *Calypte costae* formed a different cluster from *A. fuciphagus* and *Apus apus* in the phylogenetic tree while both are also in the same Apodiformes order (Figure 4). Phylogenetic analysis of *CHD‐W* showed that four (Apodiformes; Gruiformes; Passeriformes; and Pelecaniformes) out of the five orders investigated had formed four clear clusters within their orders, including the studied order: Apodiformes (Figure 5). Whereas in *CHD‐Z*, four (Accipitriformes; Columbiformes; Galliformes; and Passeriformes) out of five orders investigated formed four clear clusters within their orders, excluding the studied order (Figure 4). In addition, *A. fuciphagus* and *Apus apus* (both are Apodiformes) showed less divergence in *CHD‐W* than *CHD‐Z* (0% *c.f*. 9%). These results suggest that evolution occurred at a higher rate in males (*CHD‐Z*) as compared to females (*CHD‐W*) and might also explain why *Calypte costae* which is in the same Apodiformes order with *A. fuciphagus* showed a low percentage of similarity in male. Similarly, Mank, Axelsson, and Ellegren (2007) on predicting a fast‐Z effect by focusing on chicken and zebra finch (have female heterogamety) suggested that evolution proceeds quicker on the Z chromosome than the W chromosome, where hemizygous exposure of beneficial non‐dominant mutations increase the rate of fixation.

## 5 CONCLUSION

Molecular sexing method using P8/WZ/W sexing marker set proved to be successful in differentiating the sex of *A. fuciphagus*. The comparison of sex evolutionary pattern between *A. fuciphagus* and 14 other bird species from the same Apodiformes order and Aves class had shown that sexing gene of *A. fuciphagus* was closely related to *Apus apus* and evolution may occur at a higher rate in CHD‐Z gene (males) compared to CHD‐W gene (females). Thus, findings from this study may be utilized to facilitate further studies on sex evolution, sex ratio, and breeding management of *A. fuciphagus* as well as CHD gene.

## CONFLICT OF INTEREST

The authors declare that they have no conflict of interest.

## AUTHOR CONTRIBUTION

**Muhammad Amin Osman:** Conceptualization (equal); Data curation (equal); Formal analysis (equal); Investigation (equal); Methodology (equal); Resources (equal); Software (equal); Validation (equal); Visualization (equal); Writing‐original draft (equal); Writing‐review & editing (equal). **Sumita Sugnaseelan:** Conceptualization (equal); Data curation (supporting); Formal analysis (supporting); Funding acquisition (supporting); Investigation (supporting); Methodology (supporting); Supervision (equal); Validation (equal); Visualization (equal); Writing‐original draft (equal); Writing‐review & editing (equal). **Jothi Malar Panandam:** Conceptualization (equal); Data curation (equal); Formal analysis (equal); Funding acquisition (lead); Investigation (equal); Methodology (equal); Project administration (equal); Resources (equal); Supervision (equal); Validation (equal); Visualization (equal); Writing‐original draft (equal); Writing‐review & editing (equal). **Izza Nurul Ab Ghani:** Conceptualization (lead); Data curation (equal); Formal analysis (lead); Funding acquisition (equal); Investigation (equal); Methodology (equal); Project administration (lead); Resources (equal); Software (equal); Supervision (lead); Validation (lead); Visualization (lead); Writing‐original draft (equal); Writing‐review & editing (lead).

## Data Availability

All data associated with this manuscript were submitted to GenBank with accession numbers MH445397, MH445398, MT591397, MT591398, and MT591399.
